# Supplementary material for: Intrinsic magnetic topological insulator phases in the Sb doped MnBi2Te4 bulks and thin flakes
Source: Nat Commun. 2019 Oct 2;10:4469. doi: 10.1038/s41467-019-12485-y (PMC6775157; doi:10.1038/s41467-019-12485-y)
Supplement: Supplementary file 1 — Supplementary Information [file 41467_2019_12485_MOESM1_ESM.pdf]

Intrinsic magnetic topological insulator phases in the Sb doped $MnBi_2Te_4$ thin flakes

Chen et al.

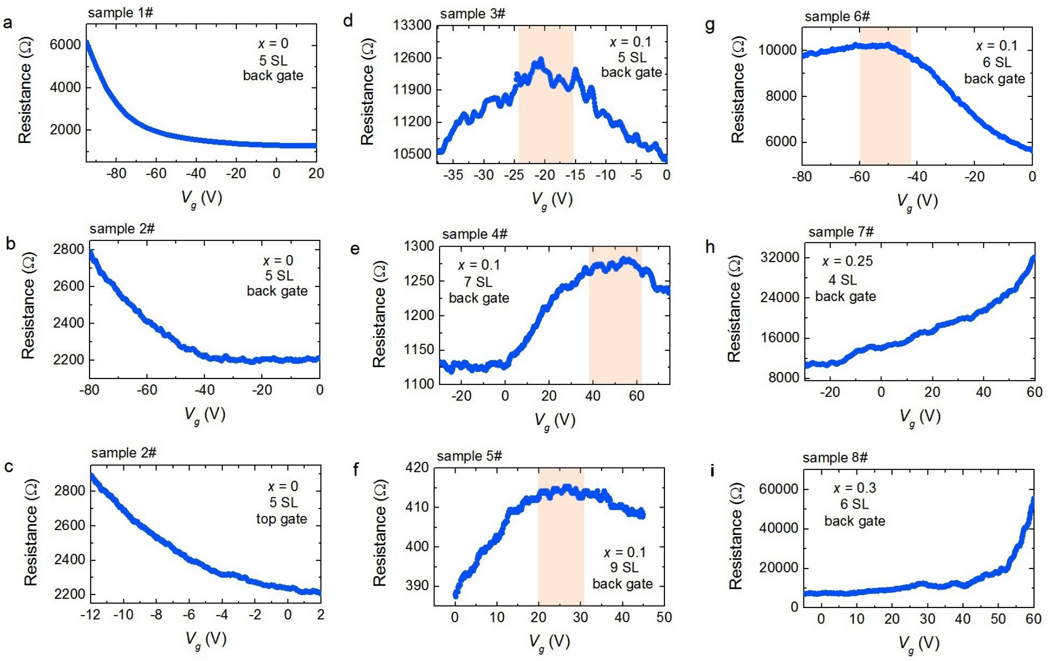

Supplementary Figure 1. Resistance versus gate voltage in different $Mn(Sb_xBi_{1-x})_2Te_4$ thin film devices. (a)-(c) $x = 0$. (d)-(g) $x = 0.1$. (h) $x = 0.25$. (i) $x = 0.3$.

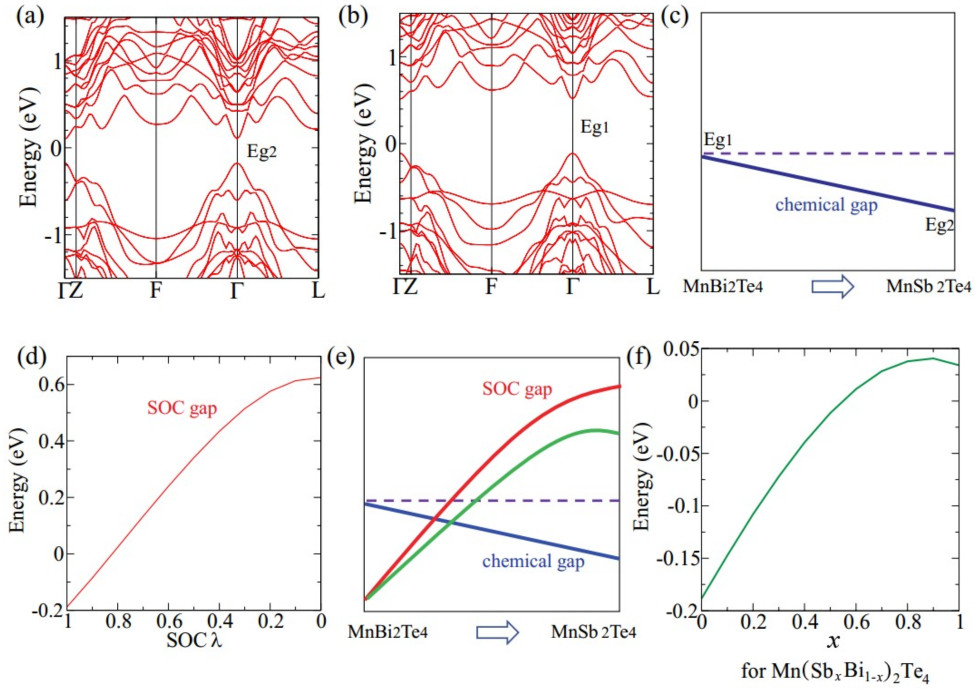

Supplementary Figure 2. Theoretical calculation of the band gap in Mn(Sb,Bi)$_2$Te$_4$. (a-b) The band structure of MnSb$_2$Te$_4$ without SOC. (b) The band structure of MnBi$_2$Te$_4$ without SOC. (c) The schematic of the chemical effect on the energy gap from MnBi$_2$Te$_4$ to MnSb$_2$Te$_4$. (d) The energy gap on the SOC effect for MnBi$_2$Te$_4$. (e) Schematically combine the SOC effect and the chemical effect. The green line is the schematic energy gap on both SOC and chemical effects. A peak of the energy gap appears. (f) The calculated energy gap for Mn(Sb$_x$Bi$_{1-x}$)$_2$Te$_4$.

Supplementary Note 1.

During the $Mn(Sb,Bi)_2Te_4$ devices fabrication and measurement, we find that the thin film device fabrication process would cause the Fermi level shifting downwards. Thus the optimized x value for thin devices is smaller than ~ 0.3. Though the specific shifted distance in each device is strongly sample-dependent, most of the pure $MnBi_2Te_4$ thin films are still heavily n-doped and hard to tune the samples to the electrical neutral point by applying gate voltage, as shown in Supplementary Figure 1(a)-(c). For thin films with $x = 0.1$, the situation changes enormously as shown in the resistance versus gate voltage curves displayed in Supplementary Figure 1(d)-(g). One can see that the electrical neutral point can be achieved under a moderate gate voltage in most of the $x = 0.1$ thin film samples (marked by pink shaded areas). Though some of the $x = 0.1$ devices are slightly n-doped while some others are p-doped because of the sample dependence of Fermi level shifting, we consider that this rate of Sb substituting ($x \sim 0.1$) is appropriate for thin devices. Further measurement in thin film devices with $x = 0.25$ and $x = 0.3$ also confirms the Fermi level shifting in $Mn(Sb,Bi)_2Te_4$ that they are heavily p-doped and hard to achieve the electrical neutral point by gate tuning (Supplementary Figure 1(h)-(i)).

Supplementary Note 2.

The energy gap goes up and then goes down, when we increase the Sb in $Mn(Sb_xBi_{1-x})_2Te_4$. We can see that there is a peak at around $x = 0.9$. We think that the origin of this peak is the competition of two effects. The first is the spin orbit coupling (SOC) effect. We know that the SOC reduces with increasing Sb. The energy gap expects to enlarge by reducing the SOC. In Supplementary Figure 2(d), we calculate the energy gap with different SOC for $MnBi_2Te_4$, which really presents that the energy gap become larger with reducing the SOC. The second is the chemical bonding effect. To see this effect, we calculate the band structures of $MnSb_2Te_4$ and $MnBi_2Te_4$ by setting the SOC to be zero, (or saying without the SOC), respectively, shown in Supplementary Figure 2(a)-(b). We can see that the energy gap of $MnSb_2Te_4$ is around 0.3 eV which is much smaller than that of $MnBi_2Te_4$ (around 0.6 eV). Therefore, the

energy gap reduces when increasing Sb, schematically seen in Supplementary Figure 2(c). So, the energy gap depends on these two effects when increasing Sb. The reducing SOC enlarges the energy gap, but the chemical effect reduce the energy gap, when increasing the Sb in $Mn(Sb,Bi)_2Te_4$, schematically seen in Supplementary Figure 2(e). Finally, the peak of the energy gap appears at $x = 0.9$, seen in Supplementary Figure 2(f).
